# High-sensitive Troponin T assay for the diagnosis of acute myocardial infarction: an economic evaluation

**DOI:** 10.1186/1471-2261-14-77

**Published:** 2014-06-13

**Authors:** Anil Vaidya, Johan L Severens, Brenda WC Bongaerts, Kitty BJM Cleutjens, Patty J Nelemans, Leonard Hofstra, Marja van Dieijen-Visser, Erik AL Biessen

**Affiliations:** 1 Department of Clinical Epidemiology and Medical Technology Assessment (KEMTA), Maastricht University Medical Centre, PO Box 5800, Maastricht 6202 AZ, The Netherlands; 2 School for Public Health and Primary Care (CAPHRI), Maastricht University, Maastricht, The Netherlands; 3 Institute of Health Policy and Management, Erasmus University Rotterdam, Rotterdam, The Netherlands; 4 iMTA - Institute of Technology Assessment, Erasmus University Rotterdam, Rotterdam, The Netherlands; 5 Department of Pathology, Cardiovascular Research Institute Maastricht (CARIM), Maastricht University Medical Centre, Maastricht, The Netherlands; 6 Cardiology Center Netherlands, Utrecht, The Netherlands; 7 Department of Clinical Chemistry, Maastricht University, Maastricht, The Netherlands; 8 Department of Epidemiology, Maastricht University, Maastricht, The Netherlands

**Keywords:** Cost-effectiveness, Decision model, Acute myocardial infarction, High-sensitive troponin T

## Abstract

**Background:**

Delayed diagnosis and treatment of Acute Myocardial Infarction (AMI) has a major adverse impact on prognosis in terms of both morbidity and mortality. Since conventional cardiac Troponin assays have a low sensitivity for diagnosing AMI in the first hours after myocardial necrosis, high-sensitive assays have been developed. The aim of this study was to assess the cost effectiveness of a high-sensitive Troponin T assay (hsTnT), alone or combined with the heart-type fatty acid-binding protein (H-FABP) assay in comparison with the conventional cardiac Troponin (cTnT) assay for the diagnosis of AMI in patients presenting to the hospital with chest pain.

**Methods:**

We performed a cost-utility analysis (quality adjusted life years-QALYs) and a cost effectiveness analysis (life years gained-LYGs) based on a decision analytic model, using a health care perspective in the Dutch context and a life time time-horizon. The robustness of model predictions was explored using one-way and probabilistic sensitivity analyses.

**Results:**

For a life time incremental cost of 30.70 Euros, use of hsTnT over conventional cTnT results in gain of 0.006 Life Years and 0.004 QALY. It should be noted here that hsTnT is a diagnostic intervention which costs only 4.39 Euros/test more than the cTnT test. The ICER generated with the use of hsTnT based diagnostic strategy comparing with the use of a cTnT-based strategy, is 4945 Euros per LYG and 7370 Euros per QALY. The hsTnT strategy has the highest probability of being cost effective at thresholds between 8000 and 20000 Euros per QALY. The combination of hsTnT and h-FABP strategy’s probability of being cost effective remains lower than hsTnT at all willingness to pay thresholds.

**Conclusion:**

Our analysis suggests that hsTnT assay is a very cost effective diagnostic tool relative to conventional TnT assay. Combination of hsTnT and H-FABP does not offer any additional economic and health benefit over hsTnT test alone.

## Background

Acute coronary syndrome (ACS) is a major cause of morbidity and mortality around the world. The most common manifestation of ACS is acute myocardial infarction (AMI). It is widely accepted that early detection and treatment of AMI has a major impact on AMI morbidity and mortality and therefore on associated costs [1-3]. According to the current guidelines, AMI is diagnosed on the basis of presenting symptoms (chest pain, shortness of breath epigastric discomfort etc.), electrocardiographic (ECG) findings and dedicated blood biomarkers of cardiac necrosis [4]. However, less than 25% symptomatic patients are finally diagnosed with AMI [5], while ECG alone may remain non diagnostic in up to 50% of cases [6]. This makes cardiac biomarker testing an important additional measure for the diagnosis of AMI.

In current clinical practice cardiac troponin T (cTnT) is the preferred biochemical marker for myocardial cell necrosis [4]. Since elevated cTnT levels are detected only 8–12 hours after onset of ischemic symptoms, the low sensitivity of cTnT assay at time of presentation is a major drawback in its use [7]. The life threatening nature of AMI and the known inconsistency in cTnT test results at its early phase lead to over-triage of patients and substantial costs to the health system [2,8].

A recently published study has concluded that high-sensitive Troponin T (hsTnT) is a useful prognostic biomarker in patients with symptoms of chest discomfort suspected for ACS [9]. Two multi centre studies have suggested that high-sensitivity Troponin assays offer superior diagnostic accuracy for the early diagnosis of AMI compared to the conventional cTnT assay [7,10]. Another AMI biomarker, heart-type fatty acid-binding protein (H-FABP), was reported to appear in the blood within one hour of myocardial necrosis and peaks after 3–4 hours [11]. Although H-FABP is not recommended as stand-alone test for diagnosis of AMI[12], combined sensitivity of cardiac troponin and H-FABP is reported to be higher than cardiac Troponin alone [13].

In this study we assessed the cost effectiveness of the hsTnT assay and combination of hsTnT (fifth generation TnT assay) and H-FABP assays for the early diagnosis of AMI in comparison with the currently in clinical practice conventional fourth generation TnT assay. To the best of our knowledge, no economic evaluation study has yet been published in Eurozone on the conventional TnT assay based diagnostic approach versus new alternatives involving hsTnT and H-FABP assays.

## Methods

### Decision analytic model tree

**Figure 1:**
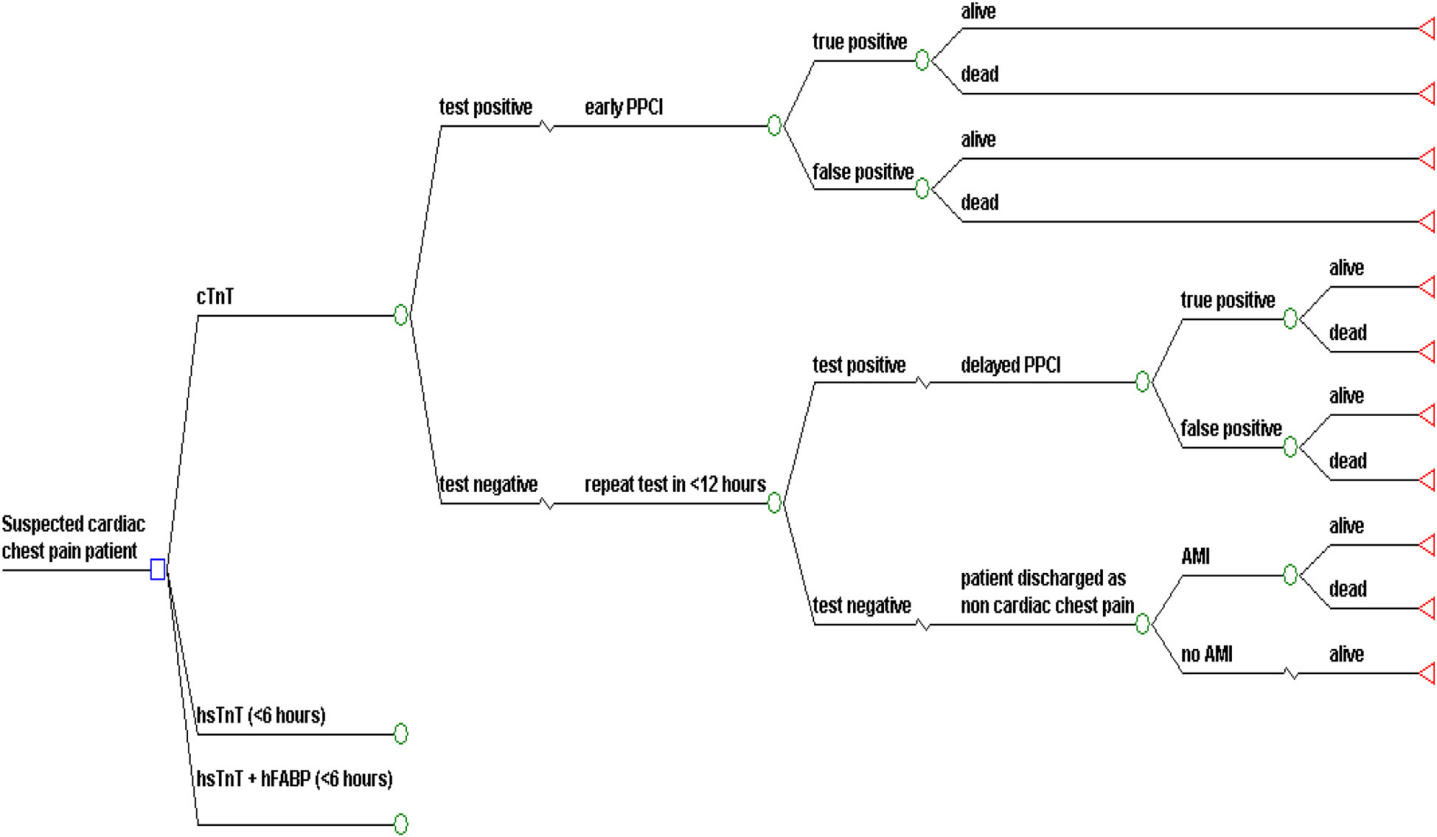
**Decision Tree Structure for Diagnosis of AMI.** Square node is decision node where patient is assigned to one of the competing strategy. Circles represent chance nodes or probabilities. Triangular terminal nodes represent the end of the paths from left to right through decision tree. Patient is discharged alive from the hospital or dies during hospitalization. Patient survives AMI lives life expectancy of AMI survivor.

This study was done in the Dutch context using a health care perspective. A decision tree was constructed to compare the costs and outcomes associated with three diagnostic strategies under evaluation in a hypothetical cohort (Figure 1).

### Diagnostic strategies

1. cTnT assay at <6 hours of symptom onset, which will be repeated after <12 hours of symptom onset, in the case of negative test result and continuing symptoms.

2. hsTnT assay at <6 hours of symptom onset, which will be repeated after <12 hours of symptom onset, in the case of negative test result and continuing symptoms.

3. hsTnT and H-FABP assays at <6 hours of symptom onset, which will be repeated after <12 hours of symptom onset, in the case of negative test result and continuing symptoms.

Correct or incorrect diagnosis of AMI and subsequent events in the model are followed for patients with chest pain presenting to the hospital. This diagnostic work up of a chest pain patient by one of the above strategies will guide the treating physician to employ the therapeutic intervention i.e. primary percutaneous coronary intervention (PPCI). PPCI is the preferred therapeutic modality to treat AMI and in The Netherlands majority of patients are treated with PPCI [14,15].

The whole process will culminate into either death or survival of the patient during hospital admission. Patient endpoint can be either alive at the end of hospitalization or dead, after presenting to hospital for suspected cardiac chest pain. For the theoretical AMI survivor an average life expectancy was assigned from the literature [16], indicating the time horizon for this modelling study to be life time. Although the exact moment in time is unknown, patients who died during the period of hospitalization were assigned a life expectancy of zero. We assumed that the diagnosis of AMI is excluded in those patients who remained negative after 2 consecutive testing.

### Model input parameters

Beside life expectancy and utility values, other parameters used in this model are probabilities of events and costs. Parameters fed into the model are computed from raw parameters obtained from the existing literature and from financial affairs department of Maastricht university medical centre (MUMC) (Table 1). The data for test accuracy of cTnT, hsTnT and hsTnT-H-FABP was determined from the diagnostic testing on the preserved blood samples from Bad Nauheim Acute Coronary Syndrome II Registry, Germany as presented by Bongaerts et al. [17].

**Table 1:** Model input parameters

**Parameter**	**Deterministic values (min – max range)**	**Distribution**	**Source**
**Costs & event occurrence**			
Cost of conventional cTnT test	€17.11(12.8-21.4)	Beta PERT	Commercial price at MUMC
Cost of new hsTnT test	€31.5 (23.6-39.4)	Beta PERT	Time & motion study*
Cost of AMI in 1^st^ year	€ 12446 ( 9334–15557)	GAMMA	[21]
Cost of AMI in subsequent year	€ 2092 (1569–2615)	GAMMA	[21]
Utility score for AMI	0.725 (0.544-0.906)	BETA	[19]
Discount rate: cost	0.4	Fixed	[23]
Discount rate: Effect	0.15	Fixed	[23]
Prevalence of AMI among patients presenting with chest pain	0.30 (0.23 -0.38)	Beta PERT	[5]
Risk adjusted mortality ratio among inappropriately discharged AMI patients	1.9 (1.43 -2.38)	Beta PERT	[20]
Life expectancy of AMI survivor (years)	8.3 (6.23-10.38)	Beta PERT	[16]
**Diagnostic accuracy**			[17]
cTnT sensitivity at ≤6 hours	0.44 (0.32-0.56)	BETA	
cTnT sensitivity at ≤12 hours	0.93 (0.85-0.97)	BETA	
cTnT specificity at ≤6 hours	0.92 (0.88-0.95)	BETA	
cTnT specificity at ≤12 hours	0.85 (0.76-0.91)	BETA	
hsTnT sensitivity at ≤6 hours	0.94 (0.87-0.98)	BETA	
hsTnT sensitivity at ≤12 hours	0.95 (0.91-0.98)	BETA	
hsTnT specificity at ≤6 hours	0.52 (0.39-0.65)	BETA	
hsTnT specificity at ≤12 hours	0.51 (0.40-0.62)	BETA	
hsTnT + hFABP sensitivity at ≤6 hours	0.97 (0.90-0.99)	BETA	
hsTnT + hFABP sensitivity at ≤12 hours	0.97 (0.93-0.99)	BETA	
hsTnT + hFABP specificity at ≤ 6 hours	0.39 (0.27-0.51)	BETA	
hsTnT + hFABP specificity at ≤ 12 hours	0.38 (0.27-0.49)	BETA	
**Event occurrence**			
Average of AMI mortality among patients given PPCI within 4 hours of presentation	0.062 (0.0468-0.0780)	Beta PERT	Calculated from [3]
AMI mortality among patients given PPCI after 4 hours of presentation	0.103 (0.077-0.1288)	Beta PERT	[3]
PPCI procedure related mortality	0.0072 (0.0054-0.009)	Beta PERT	[18]

*Time & Motion study done at Pathology Laboratory, MUMC.

### Event probability calculation

Application of Bayes’ Theorem allows us to interpret the test results. Pre-test probability or prior probability of presence/absence of a disease is calculated using Bayesian revision. Prevalence of AMI among symptomatic patients presenting at hospital and diagnostic test sensitivity and specificity are used to calculate various event probabilities. Prevalence of AMI among symptomatic patients presenting to hospital as reported in a large multi-centre study [5] is used in our model.

Post PPCI mortality among AMI patients is abstracted from the literature. Mortality increases with delay in PPCI and is reported from 15 minutes until 240 minutes delay in PPCI after presentation [3]. Average mortality, calculated from mortality reported at different points of time between 15 minutes to 240 minutes, is used in our model after initial biomarker testing at presentation. Average mortality for patients given PPCI within 4 hours of presentation is 6.24%. Mortality among patients in whom PPCI is delayed by ≥4 hours is assumed to be 10.3% based on the mortality figure reported in the literature for PPCI at 240 minutes.

Procedural mortality related to PPCI [18], post AMI life expectancy [16] utility score for AMI survivors [19] and mortality among inappropriately discharged AMI patients in comparison to hospitalized patients [20] are taken from the published literature.

### Cost calculation

The cost of diagnostic cTnT test was 17.11 Euros (data obtained from the financial affairs department of Maastricht university medical centre **(**MUMC) database. MUMC publishes standard prices of health care products available at the MUMC every year in a freely available database [21]. Cost estimates for the new diagnostic tests hsTnT/hFABP (i.e. 21.50 euro), were based on the database, consultation with experts and an in-house time and motion study performed by a senior laboratory technician at the Pathology department of the MUMC who recorded the various stages of test procedure in a time sheet. The unit costs of resources identified for performing the assay was used to calculate the total cost per diagnostic test. Costs incurred in the first year of AMI survival are higher as the primary cost driver is PPCI as a therapeutic intervention. The Dutch costs for the AMI survivors for first year and subsequent years were taken from the published literature [22]. All costs used in the model were converted to Year 2012 costs using harmonized index of consumer prices data from the Dutch bureau of statistics [23].

Longer term costs and outcomes are discounted as per the Dutch pharmacoeconomic guidelines [24].

### Outcome measures

The effectiveness of diagnostic test strategies is measured in terms of survival probabilities in AMI patients during hospitalization and incremental life years gained (LYGs) by AMI survivors. Quality adjusted life years (QALYs) are derived by multiplying LYGs with the Post AMI utility score reported in the literature [19].

### Analyses

The expected costs and outcomes of all the three strategies were calculated and incremental cost effectiveness ratios were determined. Cost effectiveness analysis(CEA) and cost-utility analysis (CUA) are approaches to compare the costs and health outcomes of a new intervention with the existing practice [25]. An incremental cost effectiveness Ratio (ICER) is calculated by dividing differential costs with differential effects between existing practice and the new health technology. When more than one, ‘new technologies’ are under evaluation then the more costly technology is compared with the less costly technology [26].

The probabilistic sensitivity analysis (PSA) considers uncertainties in all the model parameters simultaneously. Probabilistic sensitivity analysis quantifies the uncertainty in the ICER, by placing a probability distribution over parameter values. Test accuracy parameter values were varied in the full range of their reported 95% confidence interval (CI). Other parameter values were varied between 75% and 125% of their point estimates. The model parameters were assigned BETA distribution and BETA Pert distribution was used if confidence intervals or standard errors were not reported in the source literature. BETA Pert distribution is a version of the Beta distribution. The costs of AMI treatment were assigned GAMMA distribution. Probabilistic sensitivity analysis of the model parameters with 1,000 iterations using Monte Carlo simulation technique yields a range of health outcome results. Net monetary benefit (NMB) framework was applied to Monte Carlo simulation data to construct the cost effectiveness acceptability curves (CEACs). This framework offers an advantage of unambiguously sorting out the acceptability of an individual simulation trial on cost effectiveness plane, for a range of ‘willingness to pay’ values [25,27].

We performed one way sensitivity analyses to assess the degree of change in results with variation of one model input parameter value at a time. All parameters were were varied in the full range of their reported 95% confidence interval (CI) or between 75% and 125% of their point estimates.

## Results

### Base case

The expected values for the three strategies namely conventional cTnT, hsTnT and combination of hsTnT and H-FABP, regarding costs and outcomes are shown in Table 2. The constructed model predicts that when a diagnosis is made using hsTnT instead of conventional TnT, a hypothetical AMI survivor will live 0.006 years (Life Year Gain-LYG) longer and will have additional 0.004 QALYs for an incremental cost of 30.70 Euros. It should be noted here that hsTnT is a diagnostic intervention which costs only 4.39 Euros/test more than the cTnT test. The ICER generated with the use of hsTnT based diagnostic strategy comparing with the use of a cTnT-based strategy, is 4945 Euros per LYG and 7370 Euros per QALY. Combination strategy of the hsTnT assay with the H-FABP assay is the next more costly new technology and as per the decision modelling guidelines it is compared with less costly hsTnT [26]. Comparison of combination arm with hsTnT alone arm shows 0.0037 and 0.0025 additional LYGs and QALYs respectively, at an incremental cost of 55.91 Euros leading to an ICER of 15286 per LYG and 22781 Euros per QALY. Details of costs, outcomes and increments for all strategies are presented in the Table 2.

**Table 2:** Base-case results costs and effects

**Diagnostic strategies**				**Increments**		
	**cTnT**	**hsTnT**	**hsTnT + H-FABP**	**hsTnT vs cTnT**	**hsTnT + H-FABP vs cTnT**	**hsTnT + H-FABP vs hsTnT**
Discounted cost	€ 15115.62	€ 15137.2	€ 15187.74	€ 30.70	€ 86.61	€ 55.91
Discounted LYGs	2.286	2.292	2.296	0.006	0.010	0.0037
Discounted QALYs	1.657	1.662	1.664	0.004	0.007	0.0025
**ICER**						
	**Incremental cost per LYG**			**Incremental cost per QALY**		
cTnT	Reference strategy			Reference strategy		
hsTnT vs cTnT	€ 4945			€ 7370		
hsTnT + H-FABP vs cTnT	€ 8780			€ 13084		
hsTnT + H-FABP vs hsTnT	€ 15286			€ 22781		

### Probabilistic sensitivity analysis

**Figure 2:**
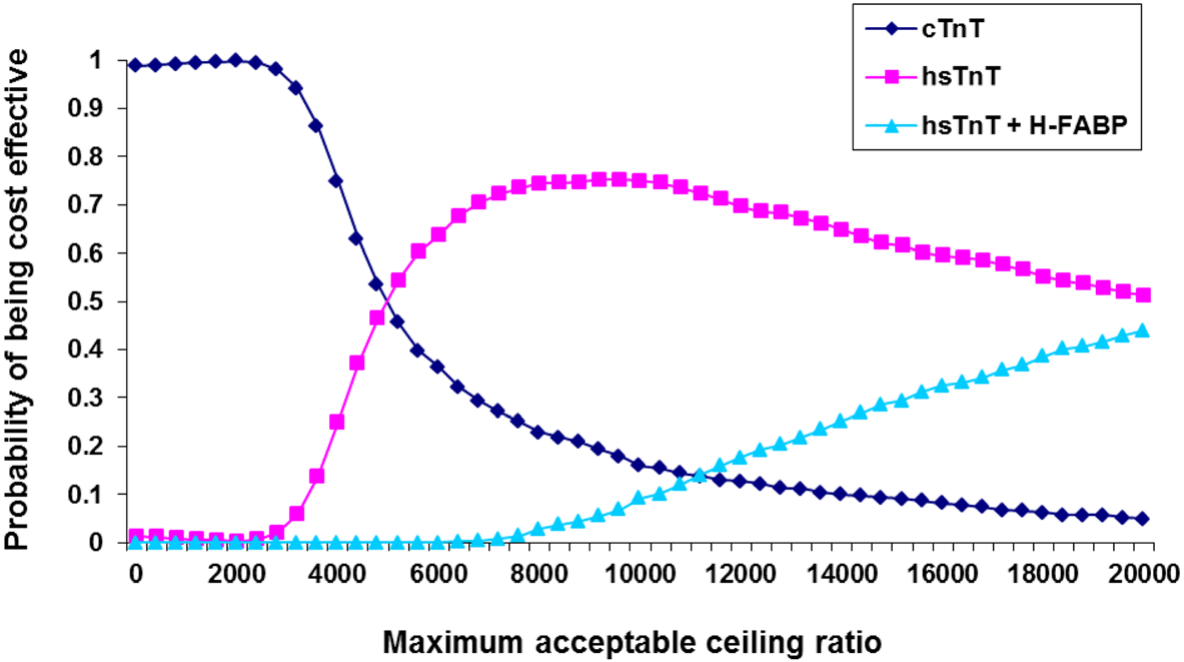
Cost effectiveness acceptability curve(s) shows likelihood that a strategy would be cost effective for a range of maximum acceptable ceiling ratios society is willing to pay for the gain of one QALY, assessed with 1000 Monte Carlo simulations.

The probabilistic results of the decision model are similar to the deterministic results hsTnT as cost effective diagnostic strategy. Figure 2 shows the probability in the PSA that each strategy is cost effective at various thresholds of willingness to pay ranging from zero to 20000 Euros. hsTnT strategy has the highest probability of being cost effective at thresholds between 8000 and 20000 Euros per QALY. The combination of hsTnT and h-FABP strategy’s probability of being cost effective remains lower than hsTnT at all willingness to pay thresholds and was not analysed further by one way sensitivity analysis.

### One way sensitivity analysis

**Figure 3:**
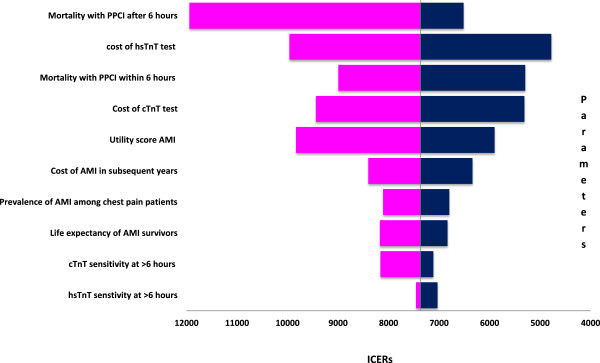
One way sensitivity analysis: Tornado diagram.

The constructed model was robust to all one way sensitivity analyses and ICER remains less than 12000 Euros per QALY which is far below the acceptable willingness to pay per QALY limit of 20000 Euros in The Netherlands. The results of one way sensitivity analyses are graphically displayed as a tornado diagram in the Figure 3. This figure shows top ten model parameters influencing the model results (ICER).

## Discussion

This modelling study assessed the cost effectiveness of two biomarker strategies for the early diagnosis of AMI i.e. hsTnT and the combination of hsTnT and H-FABP. Economic and clinical consequences of using these new tests instead of conventional cTnT test were extrapolated by decision modelling. Our findings suggested that hsTnT is a cost effective tool to diagnose AMI at the Emergency Department in patients presenting with chest pain. Use of hsTnT diagnostic assay resulted in gain of additional QALYs compared with the existing 4^th^generation diagnostic cTnT test. A combination strategy of performing the 5^th^generation hsTnT and H-FABP assays simultaneously did not bring any additional benefit and even incurred higher costs.

This study was performed with a health care payer’s perspective. The societal perspective for this study was not considered because the average age of AMI patients in The Netherlands is 66.7 years [28]. Therefore, productivity loss in this age group may not be significant and impact of this effect was not weighed or included. Indirect costs associated with time spent by family members for the care of these patients are also difficult to estimate for the societal perspective.

A potential limitation of this study is that the probabilities of clinical outcomes in our model are derived from diagnostic accuracy estimates for hsTnT and H-FABP from a single cohort with relatively small number of patients from Bad Nauheim Acute Coronary Syndrome II Registry, Germany. However, in sensitivity analysis our test accuracy data was varied between full ranges of confidence interval (CI) limits to test the robustness of the model. Application of the net monetary benefit (NMB) framework revealed the dominance of hsTnT strategy showing highest net monetary benefit for it. Furthermore, a recently published meta-analysis [29] for diagnostic accuracy of cTnT and hsTnT at the time of patient’s presentation to the emergency department has similar accuracy figures as our source article by Bongaerts et al. [17].

This study shares the general limitations of economic modelling. Complex medical practice is difficult to transform into a decision tree model. This applies to our model as well. All test positive patients will not undergo PPCI in real life situation. Repeated biomarker testing, clinical judgment and ECG findings play a crucial role in decision making on invasive intervention. Our decision tree model attempts to reflect the true clinical practice as closely as possible and the model’s robustness has been rigorously tested for changes in clinical and economic variables. All the model assumptions and uncertainties were addressed by performing one way sensitivity analysis and state-of-the-art Probabilistic Sensitivity Analysis. PSA is one of the most sophisticated methods to address uncertainty allowed systematic propagation of uncertainty in all model parameters and offered a statistical interpretation of the joint distribution of incremental costs and effects. The model outcomes (expected costs and QALYs of the strategies) were based on the results of the probabilistic sensitivity analysis (PSA) with 1,000 simulations. The results of our study are in line and are comparable to the recently published National Institute for Health Research (NIHR) UK, document showing the value of hsTnT based early diagnosis of AMI. This document also concludes that there is currently insufficient evidence to support routine use of alternative biomarkers alongside troponin [30].

Our estimation of post PPCI mortality is based on averaged mortality figures reported by Rathore et al. and covers the 15–240 min delay from diagnosis to PPCI after arrival to hospital [3]. The study by Rathore et al. [3] shows that more than 80% of patients underwent a PPCI within 120 minutes of hospital arrival, implying that the applied average mortality rate in our model may be an overestimation. Lower mortality rate will shift ICER more in favour of hsTnT and would make hsTnT even more cost effective.

Our model conforms to the principles of good practice for decision analytic models with use of transparent data and modelling technique as per the guidelines laid by the international society for pharmacoeconomics and outcome research (ISPOR) task force [31]. Strength of this model is its potential transferability as model inputs can be adapted to a new setting easily. Inter- or intra-country variations in costs, prevalence of AMI and therapeutic intervention outcomes caused by availability and accessibility of health care can be incorporated into the model. Using cost effectiveness threshold from WHO-CHOICE (world health organization – CHOosing Interventions that are Cost Effective) project for ceiling ratio in a particular country, cost effectiveness analysis can be performed for that country [32].

The newly introduced hsTnT biomarker assay significantly contributes to the early diagnosis of AMI and appears to be a promising diagnostic intervention for AMI. Although a multi-marker approach using hsTnT and H-FABP may allow an early rule out of the disease but economic modelling of cost and consequences for this combination predicts its inferiority to hsTnT alone. Moreover, in a non ST elevation AMI patients study by Giannitsis et al. showed that a doubling of the hs-TnT concentration within 3 hours with the second concentration above the 99^th^ percentile value is associated with a positive predictive value for AMI of 100% and a negative predicting value of 88% [33]. This indicates that with the hsTnT assay within 3 hours instead of the 6 hours examined in this study, a definitive outcome can be obtained.

## Conclusions

This economic evaluation concludes that hsTnT assay is a cost effective alternative for the diagnosis of AMI to the existing diagnostic marker assay in clinical practice. Combination of two biomarkers, hsTnT and H-FABP for the diagnosis of AMI does not bring any added advantage. Future replacement of cTnT with hsTnT in clinical practice is expected to save substantial health care costs and to improve Health Related Qulaity of Life among AMI patients. However, data for hsTnT and its combination with other biomarkers from further research is needed to support and strengthen the results of this modelling study.

## Abbreviations

ACS: Acute coronary syndrome; AMI: Acute myocardial infarction; ECG: Electrocardiogram; cTnT: Cardiac troponin; hsTnT: High-sensitive troponin; H-FABP: Heart type fatty acid binding protein; PPCI: Primary percutaneous coronary intervention; MUMC: Maastricht university medical centre; LYG: Life year gained; QALY: Quality adjusted life years; CEA: Cost effectiveness analysis; CUA: Cost utility analysis; PSA: Probabilistic sensitivity analysis; CI: Confidence interval; NMB: Net monetary benefit; ICER: Incremental cost effectiveness ratio; CEAC: Cost-effectiveness acceptability curve; CHOICE: CHOosing interventions that are cost effective.

## Competing interest

The authors declare that they have no competing interests.

## Authors’ contributions

Conceived and designed the economic evaluation: AV and JLS. Data: BWCB and EALB. Wrote the paper: AV, JLS, EALB. Critical revision of the manuscript: KBJMC, PJN, LH and MDV. Final approval of the manuscript for publication: JLS and EALB. All authors read and approved the final manuscript.

## Pre-publication history

The pre-publication history for this paper can be accessed here:

http://www.biomedcentral.com/1471-2261/14/77/prepub
